# The Xylanase Inhibitor TAXI-I Increases Plant Resistance to *Botrytis cinerea* by Inhibiting the BcXyn11a Xylanase Necrotizing Activity

**DOI:** 10.3390/plants9050601

**Published:** 2020-05-08

**Authors:** Silvio Tundo, Maria Chiara Paccanaro, Ibrahim Elmaghraby, Ilaria Moscetti, Renato D’Ovidio, Francesco Favaron, Luca Sella

**Affiliations:** 1 Department of Land, Environment, Agriculture and Forestry (TESAF), Research Group in Plant Pathology, University of Padova, Viale dell’Università 16, 35020 Legnaro, Italy; silvio.tundo@unipd.com (S.T.); mariachiara.paccanaro@hotmail.it (M.C.P.); ibrahim_elmaghraby@yahoo.com (I.E.); francesco.favaron@unipd.it (F.F.); 2 Agricultural Research Center, Central Laboratory of Organic Agriculture, 9, Cairo Univ. St., Giza 12619, Egypt; 3 Department of Ecology and Biology (DEB), Biophysics and Nanoscience Centre, University of Tuscia, Via S. Camillo de Lellis snc, 01100 Viterbo, Italy; ilariamoscetti@gmail.com; 4 Department of Agricultural and Forestry Sciences (DAFNE), University of Tuscia, Via S. Camillo de Lellis snc, 01100 Viterbo, Italy; dovidio@unitus.it

**Keywords:** cell wall, cell wall degrading enzymes, *triticum aestivum* xylanase inhibitor, cell death, grey mold

## Abstract

During host plant infection, pathogens produce a wide array of cell wall degrading enzymes (CWDEs) to break the plant cell wall. Among CWDEs, xylanases are key enzymes in the degradation of xylan, the main component of hemicellulose. Targeted deletion experiments support the direct involvement of the xylanase BcXyn11a in the pathogenesis of *Botrytis cinerea*. Since the *Triticum aestivum* xylanase inhibitor-I (TAXI-I) has been shown to inhibit BcXyn11a, we verified if TAXI-I could be exploited to counteract *B. cinerea* infections. With this aim, we first produced *Nicotiana tabacum* plants transiently expressing TAXI-I, observing increased resistance to *B. cinerea*. Subsequently, we transformed *Arabidopsis thaliana* to express TAXI-I constitutively, and we obtained three transgenic lines exhibiting a variable amount of TAXI-I. The line with the higher level of TAXI-I showed increased resistance to *B. cinerea* and the absence of necrotic lesions when infiltrated with BcXyn11a. Finally, in a droplet application experiment on wild-type *Arabidopsis* leaves, TAXI-I prevented the necrotizing activity of BcXyn11a. These results would confirm that the contribution of BcXyn11a to virulence is due to its necrotizing rather than enzymatic activity. In conclusion, our experiments highlight the ability of the TAXI-I xylanase inhibitor to counteract *B. cinerea* infection presumably by preventing the necrotizing activity of BcXyn11a.

## 1. Introduction

*Botrytis cinerea* Pers. is a necrotrophic fungus causing grey mold disease on several dicotyledonous plants. This fungus can infect more than 1000 plant species [1], including almost all vegetable and fruit crops. It causes severe damage, both pre- and post-harvest, with annual losses of $10 billion to $100 billion worldwide [2]. 

Genomic studies reveal that necrotrophic pathogens such as *B. cinerea* contain core functions, including cell wall degrading enzymes (CWDEs) and secondary metabolites (e.g., toxins) that support their lifestyle of killing plant cells [3]. Whether the toxic compounds secreted by *B. cinerea* induce necrosis or also trigger programmed cell death (PCD) remains to be determined. However, pieces of evidence suggest that *B. cinerea* has a short biotrophic phase during which the fungus would suppress autophagy, a PCD mechanism activated by plants as defense response after pathogen recognition. Since autophagy can stop the infection, its suppression would allow the fungus to grow and accumulate biomass in the plant tissue. After this short biotrophic stage, *B. cinerea* produces phytotoxic metabolites to activate its necrotrophic phase by inducing apoptotic cell death [4]. 

One of the *B. cinerea* virulence factors identified so far, the BcXyn11a xylanase, is an endo-β-1,4-xylanase that possesses both enzymatic and necrotizing activity and can trigger plant immunity [5,6]. Endo-β-1,4-xylanases (endo-xylanases; EC 3.2.1.8) are glycoside hydrolase enzymes able to catalyze the hydrolysis of β-1,4-xylan, an abundant structural polysaccharide particularly present in the primary cell wall of monocot plants [7]. 

Some fungal xylanases have been shown to play an important role in the pathogenesis of necrotrophic fungi, such as the SsXyl1 xylanase of *Sclerotinia sclerotiorum*, which is required for pathogenicity during infection of *Arabidopsis thaliana* [8], and the mentioned BcXyn11a, whose necrotizing activity is crucial for fungal virulence on tomato leaves and grape berries [5,6]. Frías et al. [9] identified a BcXyn11a short 25-residue peptide, named Xyn25, containing two conserved regions of four consecutive amino acid residues able to determine all the effects observed in planta with the BcXyn11a, including necrosis and activation of defense responses [9]. Therefore, it could be hypothesized that the BcXyn11a xylanase of *B. cinerea* could play a dual role during plant infection. On the one hand, this protein could be recognized by the host, which activates defense responses such as autophagic cell death. On the other hand, its secretion could induce apoptosis, activating the necrotrophic phase of the fungus.

Among the defense mechanisms used by plants to counteract microbial pathogens, xylanase inhibitors (XIs) can reduce or completely block the fungal endo-xylanolytic activity. Three inhibitor families with different inhibitory capacities have been identified in cereals and other grass species: *Triticum aestivum* XI (TAXI) [10], xylanase inhibitor protein (XIP) [11] and thaumatin-like XI (TLXI) [12]. These proteins inhibit the activity of microbial xylanases *in vitro* but are ineffective against plant xylanases, thus suggesting an important role in plant defense [13]. 

TAXI-type inhibitors are widely represented. The possibility to engineer monocot plants by overexpressing XIs has been exploited in wheat and rice obtaining plants more resistant to fungal infections [14,15,16]. In particular, the overexpression of TAXI-III (one of the members of the TAXI family) in transgenic wheat plants [14] causes a delay in Fusarium head blight (FHB) symptoms caused by *Fusarium graminearum*. During the infection process, this fungal pathogen produces two endo-xylanases, namely FGSG_03624 and FGSG_10999, which contain the two conserved regions found in the Xyn25 peptide of the BcXyn11a xylanase shown to induce necrosis and defense responses [9]. The inhibition of the hydrolytic activity and the prevention of the necrotizing activity of the *F. graminearum* xylanases by TAXI-III have been suggested as the possible mechanisms responsible for the delay of FHB symptoms [17,18]. However, the total xylanase activity is not recognized as necessary for *F. graminearum* virulence in wheat [19,20], unless the polygalacturonase activity is also compromised [21]. Thus, the mechanism underlying the increased resistance of TAXI-III wheat plants [14] is not precisely established.

TAXI-I, another member of the TAXI family, is one of the most represented XI in wheat endosperm. TAXI-I, similarly to TAXI-III, is expressed in wounded leaves, but only TAXI-III is strongly induced after microbial infection [22]. TAXI-I is a 381 amino acid non-glycosylated protein [23] that inhibits fungal and bacterial endo-xylanases [11,24,25].

The graminaceous inhibitor TAXI-I is known to inhibit the BcXyn11a enzymatic activity *in vitro* [26]. The ability of TAXI-I to reduce enzymatic and necrotizing activities of BcXyn11a in planta, and the possible effect on *B. cinerea* virulence has not been verified yet. Thus, we transiently and constitutively expressed TAXI-I in *Nicotiana tabacum* and *A. thaliana*, respectively, to investigate whether this inhibitor could be exploited in dicot plant species to increase resistance to *B. cinerea*. To decipher if TAXI-I limits *B. cinerea* infection by inhibiting the necrotizing activity of BcXyn11a, we performed cell death and H_2_O_2_ assays. To support the hypothesis that the increased resistance observed in transgenic TAXI-I plants is due to a specific interaction between TAXI-I and the BcXyn11a xylanase, and not to some other plant defense mechanisms, TAXI-I plants were checked for a possible change in disease response to the bacterial pathogen *Pseudomonas syringae* pv. *maculicola*. This bacterium does not produce any hydrolytic enzymes [27]. 

## 2. Results

### 2.1. Tobacco Plants Transiently Expressing TAXI-I Show Increased Resistance to B. Cinerea

**Figure 1:**
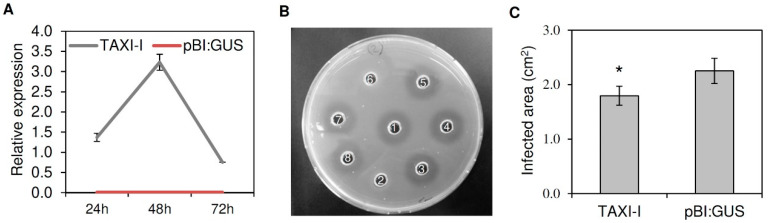
Characterization of *Nicotiana tabacum* leaves agro-infiltrated with *Triticum aestivum* TAXI-I and pBI:GUS and inoculated with *Botrytis cinerea.* (**A**) Relative expression of *TAXI-I* transcript was determined by RT-qPCR in *N. tabacum* agro-infiltrated leaves at different time points post-agro-infiltration. Relative expression was normalized with the tobacco *actin* gene set to 1. Data represent the average ± standard error of two independent experiments. (**B**) Radial gel diffusion assay to quantify the xylanase inhibition activity. A total of 70 ng of BcXyn11a were incubated alone or in the presence of 20 µg of total native or boiled protein extracts obtained from *N. tabacum* plants agro-infiltrated with TAXI-I or pBI:GUS. Samples: (1) BcXyn11a; (2) BcXyn11a + native TAXI-I extract; (3) BcXyn11a + boiled TAXI-I extract; (4) BcXyn11a + native pBI:GUS extract; (5) BcXyn11a + boiled pBI:GUS extract; (6) BcXyn11a + purified TAXI-I; (7) BcXyn11a + boiled purified TAXI-I; (8) BcXyn11a + McIlvaine’s buffer. The absence of the halo indicates the presence of inhibition activity. (**C**) Lesion area produced by *B. cinerea* (strain B05.10) on tobacco leaves expressing TAXI-I or pBI:GUS at three days post-inoculation (dpi). Bars indicate the standard error (SE) calculated from three independent infection experiments. * indicates significant differences at *p* < 0.05 applying the Student’s t-test.

To test if TAXI-I could be exploited to counteract *B. cinerea* infection, we first produced tobacco plants transiently expressing this inhibitor. Therefore tobacco plants were transformed with *Agrobacterium tumefaciens* strains harboring the pBI:TAXI-I or the empty pBI:GUS vector as a negative control. The transformed plants were subjected to RTqPCR analysis to verify the expression of the *TAXI-I* gene. Compared to the tobacco *actin* gene used as housekeeping, agro-infiltrated tobacco leaves expressing TAXI-I showed a peak of expression two days after agro-infiltration, with about a three folds increase. As expected, TAXI-I was not expressed in pBI:GUS leaves (Figure 1A). 

To verify the activity of TAXI-I, protein extracts from TAXI-I or pBI:GUS tobacco leaves were assayed for inhibition activity against the *B. cinerea* xylanase BcXyn11a (specific activity of 78 U/mg protein), which was heterologously expressed in *Pichia pastoris*. A total of 70 ng of the purified xylanase, corresponding to about 1 × 10^−6^ U of xylanase activity, were used in a cup-plate inhibition assay. Then, 20 µg of proteins, extracted three days after agro-infiltration from TAXI-I transiently expressing tobacco plants, completely inhibited BcXyn11a, while no inhibition activity was detected using the same amount of protein extracted from tobacco plants transiently expressing pBI:GUS used as a negative control (Figure 1B). Boiled total protein extracts of TAXI-I or pBI:GUS plants were also assayed against BcXyn11a, but no inhibition activity was observed, excluding the possibility of a non-proteinaceous inhibition (Figure 1B). 

Four days after agro-infiltration, tobacco plants transiently expressing TAXI-I were subjected to infection experiments with *B. cinerea*. At three days post-inoculation (dpi), average leaf lesion area of TAXI-I tobacco plants was 1.79 ± 0.17 cm^2^ while that of plants agro-infiltrated with the pBI:GUS plasmid was 2.25 ± 0.23 cm^2^, with a significant symptom reduction of about 20% in TAXI-I plants in comparison to the control plants (Figure 1C).

### 2.2. Production and Characterization of TAXI-I Transgenic Arabidopsis Lines

**Figure 2:**
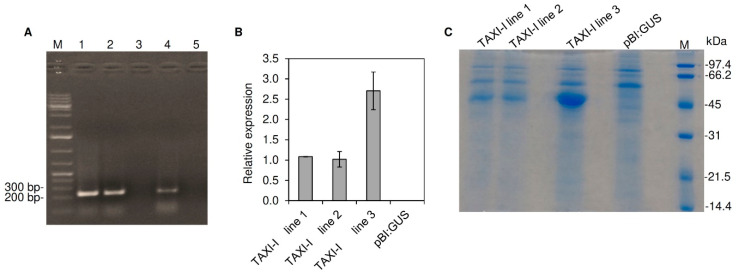
Selection and characterization of transgenic *Arabidopsis thaliana* transgenic plants transformed with the pBI:TAXI-I or pBI:GUS constructs. (**A**) PCR amplification performed using gene-specific primers and the total genomic DNA of T_0_ plants. Amplicons were separated on 1% (*w*/*v*) agarose gel. Samples: (1) TAXI-I line 1; (2) TAXI-I line 2; (3) pBI:GUS; (4) TAXI-I line 3; (5) water; M: marker. (**B**) The relative expression level of *TAXI-I* gene determined by quantitative RT-PCR (RT-qPCR) in four weeks old *A. thaliana* transgenic plants. Each transcript was normalized with the *Arabidopsis ubiquitin* gene set to 1. Data represent the average ± standard error (indicated by bars) of two RT-qPCR experiments. (**C**) SDS_PAGE analysis of extracellular fluids extracted by vacuum-infiltration from leaves of *Arabidopsis* transgenic lines transformed with the pBI:TAXI-I or pBI:GUS constructs. SDS-PAGE analysis was performed in 12% (*w*/*v*) polyacrylamide gel. M: marker.

Seeds from *Arabidopsis* plants were transformed by a floral dip with an *Agrobacterium tumefaciens* strain containing the *TAXI-I* encoding gene and were selected by plating on Murashige and Skoog’s medium supplemented with kanamycin. A PCR performed with primers specific for the *TAXI-I* gene on DNA extracted from plants with antibiotic resistance confirmed the presence of the transgene and three TAXI-I transgenic lines (TAXI-I line 1, TAXI-I line 2 and TAXI-I line 3), phenotypically identical to wild-type plants, were selected for further analysis (Figure 2A).

The expression of the *TAXI-I* gene was tested on T_2_ generation transgenic lines by RT-qPCR using the *Arabidopsis ubiquitin* gene as housekeeping. The analysis confirmed the expression of *TAXI-I* gene, with a peak of the corresponding transcript of about one fold in lines 1 and 2 and 2.7 folds in line 3 (Figure 2B). Conversely, no *TAXI-I* transcripts were detected in the pBI:GUS transgenic line.

Since the TAXI-I deduced protein contains a signal peptide of 21 residues (predicted by the SignalP software) for the secretion in the apoplast, we verified the presence of the TAXI-I inhibitor in extracellular fluids of transgenic and pBI:GUS control plants. The extracts were not contaminated by cytoplasmic material, as revealed by the absence of glucose-6-phosphate dehydrogenase activity. After SDS-PAGE, a 45 kDa band was present in the extracellular fluids of all the three TAXI-I lines while it was absent in pBI:GUS plants (Figure 2C). The protein was more abundant in the TAXI-I line 3 (Figure 2C) and was identified as TAXI-I by MALDI-TOF/TOF (Supplementary Table S1).

To determine the TAXI activity in the plant tissues, variable amounts—from 0.1 to 5 µg of total protein extracted from different leaves of the transgenic plants—were assayed against 70 ng of BcXyn11a. The TAXI-I line 3 contained about five folds more inhibitory activity than lines 1 and 2, since 0.18^A^ ± 0.01 µg of leaf protein extract from line 3 was estimated to inhibit by 50% the activity of the BcXyn11a in comparison to 0.96^B^ ± 0.03 and 0.99^B^ ± 0.07 µg of proteins needed to obtain the same level of inhibition from line 1 and 2, respectively (different letters indicate significant differences according to Tukey test; *p* < 0.01). No inhibition activity was detected by using up to 20 µg of total protein extract from *Arabidopsis* pBI:GUS plants. 

### 2.3. TAXI-I Limits B. Cinerea Infection in Planta and Inhibits the Necrotizing Activity of BcXyn11a in a Dose-Dependent Manner

**Figure 3:**
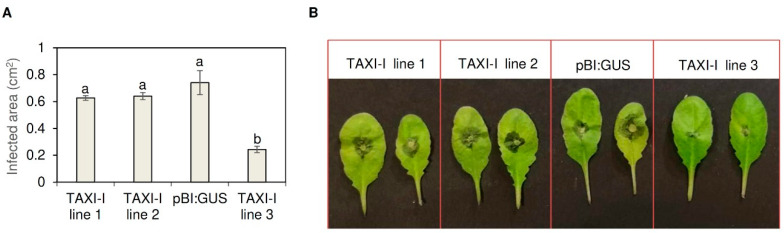
Histogram (**A**) and picture (**B**) showing the lesion area produced by *Botrytis cinerea* (strain B05.10) on *Arabidopsis thaliana* TAXI-I and pBI:GUS leaves at 48 h post-inoculation (hpi). Leaves were inoculated with disks (0.3 cm diameter) containing actively growing mycelium. Lesion areas are expressed in cm^2^ ± standard error (SE) calculated from at least three independent infection experiments, each performed with 12 plants per line. All data were subjected to ANOVA analysis. Different letters correspond to significant differences (*p* < 0.05), according to the Tukey test.

Basal *Arabidopsis* leaves of TAXI-I transgenic lines and pBI:GUS control plants were inoculated with agar disks colonized by actively growing *B. cinerea* mycelium, and the infected area was measured after 48 h from inoculation. Disease symptoms appeared as a leaf discoloration around the inoculation site; TAXI-I transgenic lines 1 and 2 showed an average infected area of 0.63 ± 0.02 cm^2^ and 0.64 ± 0.03 cm^2^, respectively, that are slightly but not significantly reduced in comparison to the area measured on pBI:GUS control plants (0.74 ± 0.09 cm^2^). Instead, the TAXI-I transgenic line 3 exhibited an average infected area of 0.24 ± 0.02 cm^2^, almost 70% lower compared to pBI:GUS plants (Figure 3A,B). 

**Figure 4:**
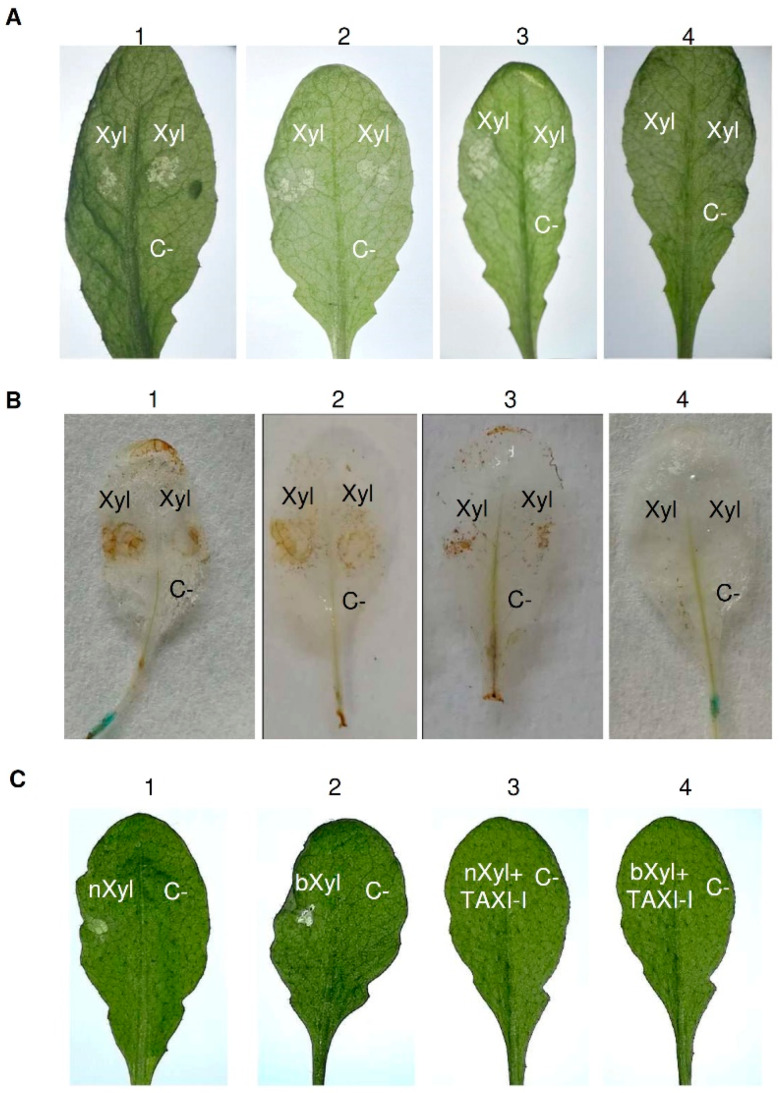
Analysis of necrosis and H_2_O_2_ production induced by BcXyn11a xylanase in *Arabidopsis thaliana* tissues and capacity of TAXI-I to limit the BcXyn11a effect in transgenic TAXI-I and wild-type *A. thaliana* (Col-0) leaves. (**A**) Necrotizing activity of BcXyn11a (Xyl) in infiltrated *A. thaliana* leaves of (1) pBI:GUS; (2) TAXI-I line 1; (3) TAXI-I line 2; (4) TAXI-I line 3 transgenic lines. (**B**) H_2_O_2_ induction by BcXyn11a xylanase in *A. thaliana* TAXI-I and pBI:GUS transgenic lines. Leaves were infiltrated with the BcXyn11a xylanase (Xyl) and treated with diaminobenzidine to reveal H_2_O_2_ accumulation. Samples: (1) pBI:GUS; (2) TAXI-I line 1; (3) TAXI-I line 2; (4) TAXI-I line 3. (**C**) Necrotizing activity of BcXyn11a assayed by droplet application method on wild-type *Arabidopsis* leaves using 70 ng of BcXyn11a (Xyl) alone or in combination with one µg of purified TAXI-I. Samples: (1) native BcXyn11a (nXyl); (2) boiled BcXyn11a (bXyl); (3) native BcXyn11a (nXyl) co-incubated with TAXI-I; (4) boiled BcXyn11a (bXyl) co-incubated with TAXI-I. In all experiments, acetate buffer 25 mM pH 5.2 (BcXyn11a buffer) was used as the negative control (C-). Xyl and C- indicates the infiltration and inoculation points. Pictures were taken three days after inoculation.

Afterward, we investigated if the capacity of the *Arabidopsis* TAXI-I transgenic line 3 to reduce *B. cinerea* infection was related to the containment of BcXyn11a necrotizing activity. To this aim, the abaxial leaf surface of the three TAXI-I transgenic lines exhibiting different levels of enzymatic inhibitory activity against BcXyn11a was infiltrated with the purified BcXyn11a at a concentration of 70 ng/μL. Three days later, the leaves of pBI:GUS, TAXI-I line 1 and TAXI-I line 2 showed necrotic symptoms (Figure 4A). Conversely, TAXI-I line 3 plants did not highlight the necrotic effect caused by BcXyn11a (Figure 4A). The capacity of this xylanase to induce the release of reactive oxygen species (ROS), one of the first signals involved in hypersensitive response (HR), was also investigated by determining the production of H_2_O_2_ in the infiltrated leaves. After 16 h from BcXyn11a infiltration, the assay highlighted the production of H_2_O_2_ in the leaves of pBI:GUS and TAXI-I lines 1 and 2 but not in the leaves of the TAXI-I line 3 (Figure 4B). To understand if the capacity of the highly expressing TAXI-I line 3 to limit the necrotizing activity of BcXyn11a could be ascribed to sequestration of the enzyme by the TAXI inhibitor, we performed experiments by treating leaves of wild-type *Arabidopsis* plants with the xylanase and the purified TAXI-I mixed. To this aim, 70 ng of BcXyn11a xylanase alone or mixed with one µg of TAXI-I, an excess amount capable to completely inhibit the enzymatic activity, were dropped on the adaxial leaf surface. Results showed that the necrosis induced on leaves by BcXyn11a was prevented if TAXI-I was present in the mixture. Necrosis was induced in plant tissues even if BcXyn11a was denatured by boiling (Figure 4C), and TAXI-I prevented the necrotizing activity of this inactive xylanase. 

Finally, to support more convincingly that the ability of TAXI-I to prevent the cell death is due to a specific interaction with the xylanase, leaves of *Arabidopsis* wild-type plants were treated with a polygalacturonase from *Sclerotinia sclerotiorum* that is not inhibited by TAXI-I. This enzyme, previously named PG4 or PGb [28], is known to induce cell death in plant tissues [29]. Leaves were treated with 70 ng of PGb both in the presence and in the absence of one µg of TAXI. After three days, in both cases, the leaves displayed necrotic symptoms (Supplementary Figure S1). 

### 2.4. TAXI-I Does Not Provide Resistance to P. Syringae pv. Maculicola

To exclude that the protection provided by TAXI-I is caused by an alteration of the plant defense system, TAXI-I transgenic plants were subjected to infection experiments with the bacterial pathogen *P. syringae* pv. *maculicola,* known to be a non-producing hydrolytic-enzymes pathogen [27]. Leaves of *Arabidopsis* transgenic lines were infiltrated with the pathogen, and the analysis of symptoms did not highlight any difference in the size of lesion area neither among the transgenic plant lines nor with the pBI:GUS plants (Figure S2). 

## 3. Discussion

Deconstruction of plant cell wall components and the killing of the host cells are the two main mechanisms responsible for the pathogenesis of the necrotrophic fungus *B. cinerea* [30,31,32,33,34,35]. Two polygalacturonases (PGs) and the BcXyn11a endo-xylanase of this fungus are reported as virulence factors [5,36,37]. The enzymatic degradation and the necrotizing activities coexist in these enzymes [6,37]. In BcXyn11a the necrotizing effect prevails upon the enzymatic activity, as demonstrated by comparing the pathogenicity of strains with catalytically active and not active enzymes [6].

The evidence that the wheat TAXI-I inhibits the BcXyn11a xylanase *in vitro* [26] prompted us to investigate whether TAXI-I could be exploited to reduce symptoms caused by *B. cinerea* in planta. In the present paper, we verified that extracts of tobacco leaves transiently expressing TAXI-I inhibit the enzymatic activity of BcXyn11a. The transformed tobacco plants also demonstrated a significant reduction of *B. cinerea* symptoms, providing evidence that the expression of TAXI-I hampers the infection of this fungus.

To confirm the results obtained by transient expression and to further investigate the interaction between BcXyn11a and TAXI-I, we constitutively expressed this protein with a signal peptide for localization in the *Arabidopsis* apoplast. Interestingly, despite proteins with an amino acid sequence similar to TAXIs that have been identified in its genome [23], we did not detect xylanase inhibitory activity in wild-type *Arabidopsis* plants. These proteins correspond to putative extracellular dermal glycoprotein precursors similar to xyloglucan-specific fungal endoglucanase inhibitors (XEGIPs) [23], which are likely not effective against endo-xylanases. As expected, we confirmed the presence of TAXI-I in the extracellular fluids of the three obtained transgenic lines and verified a markedly higher amount of TAXI-I protein and inhibitory activity in the extracts from the transgenic line 3 compared to transgenic lines 1 and 2. Infection experiments showed that only plants of the TAXI-I line 3 were more resistant to *B. cinerea* infection in comparison to the control plants, thus suggesting that the protection effect displayed by TAXI-I is dose-dependent. In support of this hypothesis, we previously demonstrated that a high level of xylanase inhibitory activity is necessary to determine a delay of *F. graminearum* symptoms development in wheat transgenic plants expressing TAXI-III [14]. To our knowledge, this is the first evidence for the possibility to engineer dicot plants with graminaceous inhibitors to increase resistance against plant pathogens.

Considering that BcXyn11a xylanase is known to contribute to *B. cinerea* virulence mainly by its necrotizing activity rather than by its enzymatic activity [6], we verified whether TAXI-I transgenic plants inhibit the necrotizing activity of the xylanase after leaf infiltration. We observed that BcXyn11a induced necrotic lesions and ROS accumulation in *Arabidopsis* leaves and that only the highly expressing TAXI-I transgenic line 3 limits these events. These findings also indicate that the inhibition of the BcXyn11a necrotizing activity depends on the amount of TAXI-I present in the plant tissue. Thus, the annulment of the xylanase necrotizing activity and the restriction of *B. cinerea* infection appear intrinsically connected. Whether the resistance exhibited by TAXI-I line 3 plants is also related to a reduction of fungal biomass accumulation remains to be clarified. In wheat expressing TAXI-III, a reduction of *F. graminearum* mycelium growth in caryopses was detected. Thus, those plants showed a decrease in both symptoms and fungal biomass compared to control plants non-expressing the xylanase inhibitor [14]. 

The reduction of the disease symptoms is likely consequent to the specific sequestration of BcXyn11a secreted by *B. cinerea* by TAXI-I present in the transgenic plants. The evidence that TAXI-I prevents the necrosis induced by the xylanase of *B. cinerea* when the two molecules are dropped together on the leaf surface of *Arabidopsis* wild-type plants corroborates this hypothesis. Moreover, the finding that TAXI-I prevents the necrotizing activity of the boiled BcXyn11a confirms previous data showing that the xylanase necrotizing activity is independent of the enzymatic activity [6,19] and that TAXIs prevent the necrosis of the inactive enzyme [17,18]. On the contrary, we did not observe the same effect when the endo-polygalacturonase (endo-PG) PGb from *Sclerotinia sclerotiorum* was mixed with TAXI-I. The endo-PG enzyme does not interact with TAXI-I, while it forms a molecular complex with plant PGIP [38]. Indeed, a soybean PGIP interacting with the cited PGb hampers the programmed cell death induced by this enzyme on soybean cells [29]. 

The inability of TAXI-I to enhance resistance against *P. syringae* also suggests that the effect provided by this inhibitor is due to its specific interaction with BcXyn11a and not to changes of other components of the plant defense signaling network. This hypothesis is also supported by the evidence that hydrolytic enzymes are not secreted by *P. syringae* pathovars [27].

How wheat TAXI-I prevents the necrotizing activity of BcXyn11a can be inferred by the location of the 25-residues necrotizing peptide in the 3D-model of BcXyn11a [9]. The peptide is near the catalytic site of the enzyme, and TAXI-I, being a competitive inhibitor, could hide this necrotizing peptide preventing its recognition by a putative plant receptor. Currently, a xylanase receptor activating hypersensitive response in plant cells has been identified in tomato [39]. 

The role of TAXI-I has been so far associated only with physiological roles in wheat growth and development. Indeed, *TAXI-I* expression is induced by wounding but not after pathogen infection [22]. Our results demonstrate that a high amount of TAXI-I in the plant tissue can increase resistance to *B. cinerea*. Being the necrotizing activity of BcXyn11a relevant to *B. cinerea* virulence, we relate the increased resistance to the inhibition of the xylanase necrotizing activity.

## 4. Materials and Methods

### 4.1. Cloning, Heterologous Expression and Purification of BcXyn11a

Total RNA was extracted from tobacco leaves infected for three days with *B. cinerea* (strain B05.10) using the RNeasy Plant Mini Kit (Qiagen Italia, Milan, Italy) according to the manufacturer’s instructions. RNA was quantified by spectrophotometric analysis, and its purity was assessed on denaturing gel. Treatment with DNaseI (Promega Italia, Milan, Italy) was performed following the manufacturer’s instructions. Reverse transcription was performed by mixing 500 ng of an oligo-dT (15/18 thymine) reverse primer with 0.5 μg target RNA and by using the ImPromII reverse transcriptase (Promega Italia s.r.l., Milan, Italy), following manufacturer’s instructions. cDNA was amplified with the primers pair BcXyn11F and BcXyn11R (designed according to BcXyn11a Accession XM_024691683.1; Supplementary Table S2) by using the “REDTaq ReadyMix PCR Reaction Mix” (Sigma-Aldrich s.r.l., Milan, Italy). The PCR was performed by repeating for 35 times the following cycle: 30 s at 94 °C; 30 s at 51 °C; 1 min at 72 °C. Amplification products were purified by using the Wizard^®^ SV Gel and PCR Clean-Up System (Promega Italia s.r.l., Milan, Italy), cloned into the pGEM-T Easy Vector (Promega Italia s.r.l., Milan, Italy) and checked by nucleotide sequencing (BMR genomics, Padova, Italy). Afterward, the cloned cDNA was re-amplified with the primers pair BcXyn11F + A and BcXyn11R + A (Table S2) containing adaptors for *EcoR*I and *Xba*I recognition sequences by using previous PCR conditions but an annealing temperature of 58 °C. The PCR amplicon, purified as above reported, was ligated into the *EcoR*I and *Xba*I sites of the pPICZαA expression vector (Thermo Fisher Scientific, Milan, Italy) and the ligation mixture was then used to transform *E. coli* competent cells. Transformed colonies were selected in Low Salt Lysogeny Broth (LBLS) medium supplemented with 25 µg/mL zeocin. Plasmid extraction, linearization, precipitation, transformation of *P. pastoris* competent cells and protein expression were performed according to Sella et al. [20]. After 96 h, liquid cultures were centrifuged at 10,000 *g* for 10 min, and supernatants were assayed for xylanase activity by radial diffusion assay [40]. The culture supernatant containing BcXyn11a protein was subjected to a two-steps precipitation with ammonium sulfate. In the first step, ammonium sulfate was added to a saturation of 45% (*w*/*v*) at 4 °C overnight. After centrifugation at 8000 *g* for 20 min, the supernatant was used for a second precipitation step with ammonium sulfate at 80% (*w*/*v*) at 4 °C overnight, and the new pellet was re-suspended in distilled water. After overnight dialysis against distilled water at 4 °C, the protein was subjected to SDS-PAGE performed in 12% (*w*/*v*) polyacrylamide gel (Figure S3). The SDS-PAGE showed the presence of two distinct bands, both belonging to the expressed BcXyn11a protein as confirmed by MALDI-TOF/TOF analysis and previously reported by Noda et al. [6]. The protein concentration was determined using the Bio-Rad protein assay kit [41] with bovine serum albumin as a standard. MALDI-TOF/TOF analysis for protein identification was performed as reported in Bertacco et al. [42]. BcXyn11a protein was afterward applied on a HiTrap Capto S 1 ml column (GE Healthcare, Sweden), equilibrated with sodium acetate buffer 50 mM pH 4.2, and eluted with 30 mL of a linear gradient of NaCl (0–0.5 M) dissolved in the same buffer. The eluted fractions were collected, dialyzed against sodium acetate buffer 25 mM pH 5.2 and assayed for xylanase activity by DNS assay, as later described. The purified protein concentration was determined by measuring the A280, using BSA as a standard. Fractions were also subjected to SDS-PAGE, as previously described. 

### 4.2. Production of the TAXI-I Construct 

The *TAXI-I* gene (Accession AJ438880.1) was amplified by PCR from the genomic DNA of *Triticum durum* cv. Svevo with the primers pair TAXI-I_1F_BamHI/TAXI-I_1259R_SacI (Table S2) by using the “REDTaq ReadyMix PCR Reaction Mix” (Sigma-Aldrich s.r.l., Milan, Italy). The PCR was performed by repeating for 35 times the following cycle: 30 s at 94 °C; 30 s at 55 °C; 90 s at 72 °C. The amplified DNA fragment was purified, and the expression cassette was put under the control of the constitutive CaMV 35S promoter and NOS terminator in the pBI vector containing the *NPTII* gene for kanamycin selection. Empty pBI:GUS vector used as a negative control was from Takara bio (Kusatsu, Japan). After verification of the correctness of all cloned sequence by nucleotide sequencing, the recombinant plasmid was transferred to *E. coli* strain DH5α grown in Lysogeny Broth (LB) medium at 37 °C and then electroporated at 2.5 KV into the *Agrobacterium tumefaciens* (strain GV3101, resistant to rifampicin and gentamicin) as described by Mozo and Hooykaans [43]. 

### 4.3. Tobacco and Arabidopsis Agrobacterium-Mediated Transformation

*Agrobacterium*-mediated transformation of *N. tabacum* (ecotype SR1) and *A. thaliana* (ecotype Col-0) was performed by agro-infiltration and floral dip methods, respectively.

For agro-infiltration of *N. tabacum* leaves, *A. tumefaciens* strains harboring *TAXI-I* or the pBI:GUS vector as control were grown overnight in a shaker at 28 °C and 200 rpm in LB supplemented with 50 μg mL^−1^ rifampicin, kanamycin and gentamicin. The overnight cultures were collected by centrifugation at 5000 *g* for 15 min at room temperature and resuspended in infiltration buffer (10 mM MgCl_2_ and 150 μg/mL acetosyringone) to a final OD_600_ of 0.8–1 [44]. The cell suspension was loaded into a 1 mL plastic syringe without needle and infiltrated into the abaxial leaf surface of 6 weeks-old *N. tabacum* plants. In particular, four leaves were infiltrated for each plant, and each leaf was infiltrated in one side of the mid-vein with the *A. tumefaciens* strain harboring *TAXI-I* and in the other side with the one harboring pBI:GUS as control. Infiltration spots were outlined with a black marker for tissue sampling. Tobacco plants were covered with transparent plastic bags and maintained at the growth conditions reported below. Infiltrated leaves were collected after 24, 48 and 72 h from agro-infiltration for expression analysis.

For *Agrobacterium*-mediated transformation by floral dip method, the *Agrobacterium* strains containing the recombinant vectors were grown overnight in LB medium supplemented with kanamycin (50 μg/mL) at 28 °C and 250 rpm. At a final OD_600_ of 0.8–1.0, 5 mL of each culture were added to 40 mL of dipping solution 5% (*w*/*v*) sucrose and 0.05% (*v*/*v*) Silwet L-77 [45]. Secondary flowering stems of *Arabidopsis* plants (10–15 cm in length) were dipped for 10 s by bending the inflorescences into the Falcon tube containing the *Agrobacterium* suspension. Then, plants were covered for 24 h and were allowed to grow under normal conditions as described below. Recovered T_0_ seeds were sterilized by immersion in 70% (*v*/*v*) ethanol for 2 min and 1% (*v*/*v*) sodium hypochlorite for 10 min, washed three times with sterile water and plated on Murashige and Skoog’s medium containing 50 μg/mL kanamycin, 1X vitamins (Sigma-Aldrich s.r.l., Milan, Italy), 3% *w*/*v* sucrose and 0.9% *w*/*v* bacto agar (Difco Laboratories, Detroit, USA). Afterward, plants were self-pollinated until T_2_ generation for subsequent experiments.

After 15 days, kanamycin-resistant seedlings were transferred to soil. Three TAXI-I transgenic lines were obtained and tested by PCR using the primer pair TAXI-I_774F/TAXI-I_1002R indicated in Table S2. The genomic DNAs of the kanamycin-resistant transformed *Arabidopsis* plants were extracted by using the “DNeasy Plant MiniKit” (Qiagen Italia, Milan, Italy) according to the manufacturer’s instructions. The PCR reaction consisted of 3 min at 94 °C, followed by 35 cycles of 94 °C for 30 s, 54 °C for 30 s and 72 °C for 3 min. 

### 4.4. RNA Extraction, cDNA Preparation and Gene Expression Analyses 

RNA was extracted from 100 mg of frozen infiltrated tobacco leaves and transgenic *Arabidopsis* leaves by using the “RNeasy Plant Mini Kit” (Qiagen Italia, Milan, Italy) following the manufacturer’s instructions. DNase treatment and reverse transcription were performed as above reported. 

Expression analysis of *TAXI-I* gene in *Arabidopsis* and tobacco leaves was carried out by qRT-PCR with the Rotor-Gene Q 2plex. The 20 µL reaction mixture contained 10 μL of 2X Rotor-Gene SYBR Green PCR Master Mix (Qiagen Italia, Milan, Italy), 0.4 mM of each specific primer (TAXI-I 774F/TAXI 1002R; Table S2) and 3 μL of cDNA as template. RT-qPCR conditions were 20 s at 94 °C, 20 s at 54 °C, 30 s at 72 °C for 40 cycles. Results were analyzed by using the Rotor-Gene 2.0.3.2 Software version (Qiagen Italia, Milan, Italy). The reference genes used in the RT-qPCR analysis were *Arabidopsis ubiquitin* (AY139810.1) and tobacco *actin* (U60495.1) genes, respectively. Two independent RT-qPCR experiments were performed with different RNA preparations.

### 4.5. Plant and Fungal Growth Conditions and Plant Infection Assays

*A. thaliana* and *N. tabacum* plants were grown in a controlled environment at 20–22 °C with a 16 h photoperiod. 

Infection experiments were performed with the fungal pathogen *Botrytis cinerea* (strain B05.10) grown on potato dextrose agar (PDA; Difco Laboratories, Detroit, USA) at 28 °C. Infection experiments of agro-infiltrated tobacco leaves with *B. cinerea* were performed by inoculating the marked infiltrated area with actively growing mycelium disks (0.5 cm diameter disks). Inoculations were performed four days after agro-infiltration (dai). Lesions were monitored for three days and the symptomatic area was measured after three days by taking a picture of the leaf surfaces with a Panasonic Lumix DMC-FZ2000 and measuring the size of symptomatic lesions with the graphic software “Fiji imageJ” (licensed under the GNU General Public License). Three independent infection experiments with at least 10 different plants for each transformant were performed.

Transgenic *Arabidopsis* leaves were inoculated on the adaxial leaf surface with fresh *B. cinerea* mycelium (0.3 cm diameter disks), as reported by Morcx et al. [46] with slight modifications. Infected plants were kept at approximately 100% relative humidity, 16 h daylight and incubated at 22 °C. Disease symptoms were monitored for two days and assessed at two days post-inoculation (dpi), as reported above. Three independent infection experiments with at least 12 different plants for each genotype were performed.

For infection experiments of transgenic *Arabidopsis* leaves with *P. syringae* pv. *maculicola*, bacterial cells were grown in King’s B medium at 28 °C O/N, collected by centrifugation at 5000 *g* for 15 min at room temperature and resuspended in MgSO_4_ 0.01 M to an OD_600_ of 0.2. The cell suspension was loaded into a 1 mL plastic syringe without a needle and infiltrated into the abaxial *Arabidopsis* leaf surface. Disease symptoms were monitored for three days and assessed at three dpi by measuring the symptomatic area as described above. Two independent infection experiments with at least 12 different plants for each genotype were performed. 

### 4.6. Extraction and Purification of TAXI-I and Enzymatic Assays

For protein extraction, samples of frozen TAXI-I tobacco agro-infiltrated and transgenic *Arabidopsis* leaves were ground in McIlvaine’s buffer pH 5.0 (0.2 M Na_2_HPO_4_, 0.1 M citric acid), shaken 1 h at 4 °C and centrifuged twice at 8000 *g* for 10 min. Supernatants were tested in radial diffusion assay [40] or by using the DNS assay described by Miller [47] and modified by Bailey et al. [48].

TAXI-I was purified from *Arabidopsis* transgenic plants expressing TAXI-I by affinity chromatography, as reported in Moscetti et al. [14]. SDS-PAGE performed in 15% (*w*/*v*) polyacrylamide gel showed the presence of a single band of approximately 45 kDa (Figure S4). MALDI-TOF/TOF analysis, performed as above reported, confirmed the identity of the protein. Purified TAXI-I was re-suspended in sodium acetate buffer 25 mM pH 5.0.

Extracellular fluids were collected from *Arabidopsis* 6-week-old rosette leaves, according to Lionetti et al. [49] with slight modifications. Briefly, leaves were stacked in the bottom of a 10-mL plastic syringe, washed with McIlvaine’s buffer pH 5.0 (0.2 M disodium hydrogen phosphate and 0.1 M citric acid) for 5 min and then vacuum-infiltrated for 10 min with the same buffer. Extracellular fluids were recovered by centrifuging the vacuum-infiltrated leaves at 500 *g* for 5 min at 4 °C. Contamination of extracellular fluids by cytoplasmic components was ruled out by measuring glucose-6-phosphate dehydrogenase activity, according to Takahama [50]. Afterward, extracellular fluids were desalted by PD10 columns (GE Healthcare Life Science, Chicago, USA) against 50 mM ammonium acetate pH 7.0 and used in SDS-PAGE analysis. MALDI-TOF/TOF analysis for protein identification was performed as reported in Bertacco et al. [42].

DNS assay was performed by incubating 70 ng of the xylanase BcXyn11a alone or with the total protein extracts (0.1–5 µg) of *Arabidopsis* transgenic leaves with beech-wood xylan (Sigma-Aldrich s.r.l., Milan, Italy) as a substrate (final concentration 0.5% *w*/*v*) at 30 °C for 15 min. The absorbance of samples was measured at 545 nm using monomeric D-xylose as a standard. One unit (U) of xylanase activity was defined as the amount of enzyme required to release 1 µmol of xylose in 1 min under the assay conditions.

Radial gel diffusion assay was performed as reported in Kalunke et al. [40] with slight modifications. Briefly, plates containing agarose (1% *w*/*v*) and birch-wood xylan (1% *w*/*v*, Sigma-Aldrich s.r.l., Milan, Italy) dissolved in McIlvaine’s buffer (pH 5) were prepared with 0.5 cm diameter wells. A total of 70 ng of the BCXyn11a xylanase alone or in the presence of 20 µg of total protein extracts from tobacco agro-infiltrated leaves were then loaded into the wells. The final reaction volume was adjusted to 50 µL by adding McIlvaine’s buffer, and plates were incubated at 30 °C for 16 h. The halo caused by enzymatic activity was visualized by adding 95% ethanol to the plates.

### 4.7. Necrosis and H_2_O_2_ Production in Arabidopsis Leaves

Necrosis on transgenic *Arabidopsis* plants was assayed by infiltration into four-weeks old leaves. BcXyn11a xylanase at a concentration of 70 ng/μL was infiltrated through stomata into the abaxial *Arabidopsis* leaf surface with a 1-mL syringe without a needle. The necrotic lesions were observed after three days. Three independent experiments with five leaves per line were performed.

The production of H_2_O_2_ was detected as reported in Quarantin et al. [51]. Briefly, four hours after infiltration, *Arabidopsis* leaves were detached and submerged in a solution of 1 mg/mL diaminobenzidine (DAB; Sigma-Aldrich s.r.l., Milan, Italy) pH 3.8. After incubating for 16 h at 22 °C in the dark, leaves were boiled for 5 min in a solution of ethanol, acetic acid and glycerol (1:1:1) to eliminate chlorophyll. Three independent experiments with five leaves per line were performed. 

Acetate buffer 25 mM pH 5.2 (BcXyn11a buffer) was used as the negative control in necrosis and H_2_O_2_ experiments.

The necrotizing activity of the purified BcXyn11a (70 ng), alone or after 10 min co-incubation with 1 µg of the purified TAXI-I, was also assayed by droplet inoculation of the adaxial surface of wild-type *Arabidopsis* leaves with 2 µL of the mixture. Three independent experiments with five plants were performed. The lesions were observed after three days. Necrosis experiments with *Sclerotinia sclerotiorum* PG4 polygalacturonase (70 ng; Zuppini et al. [29]) were performed by inoculation as described above. 

### 4.8. Statistical Analysis

*Arabidopsis* infection and DNS inhibition data were subjected to ANOVA by using SYSTAT12 software (Systat Software Incorporated, San Jose, CA, USA). When significant F values were observed, a pairwise analysis was carried out by the Tukey Honestly Significant Difference test (Tukey test) at *p* < 0.05 confidence level. Tobacco infection data were statistically analyzed applying the Student’s t-test.

## 5. Conclusions

Generating new knowledge on protein-protein interaction is an opportunity to enhance our understanding of the host–pathogen relationship. Studying these interactions is essential to fully understand and predict the effect of a particular plant defense mechanism against pathogenic microorganisms. To date, there is no commercial resistant transgenic cultivar available against *B. cinerea* [52]. Our results indicate the feasibility of increasing plant resistance to *B. cinerea* by exploiting the protein inhibitor TAXI-I. This inhibitor counteracts the necrotizing activity of the BcXyn11a xylanase, a well-known virulence factor of *B. cinerea*. To our knowledge, this is the first demonstration that a graminaceous inhibitor can be used in dicot plant species to counteract a fungal pathogen.

## Supplementary Materials

The following are available online at https://www.mdpi.com/2223-7747/9/5/601/s1. Figure S1: necrotic symptoms in *Arabidopsis thaliana* (Col-0) leaves inoculated with a purified polygalacturonase from *Sclerotinia sclerotiorum* (PGb) alone or in combination with the purified TAXI-I. Pictures were taken three days after inoculation. Water was dropped as the negative control (C-); Figure S2: infection experiments with *Pseudomonas syringae* pv. *maculicola* on transgenic TAXI-I and pBI:GUS lines. (A) Infected leaf area produced by *P. syringae* pv. *maculicola* infiltrated on leaves of transgenic and pBI:GUS lines at 3 dpi. Infected leaf areas, calculated with the graphic software “Fiji imageJ”, are expressed in cm^2^ ± standard error (SE) calculated from at least two independent infection experiments. All data were subjected to ANOVA analysis. Letters correspond to the ranking of Tukey test at *p* < 0.05. (B) Characteristic symptoms produced by *P. syringae* pv. *maculicola* on *Arabidopsis* TAXI-I and pBI:GUS transgenic lines at 3 dpi; Figure S3: SDS-PAGE analysis of the BcXyn11a xylanase protein heterologously expressed in *Pichia pastoris* and purified through HiTrap Capto S 1 column. (M) Molecular mass marker; (1) BcXyn11a; (2) BcXyn11a buffer; Figure S4: SDS-PAGE analysis of TAXI-I protein inhibitor purified by affinity chromatography on an *Aspergillus niger* endo-1,4-β-xylanase M4-sepharose conjugated column. (M) Molecular mass marker; (1) purified TAXI-I; Table S1: MALDI-TOF/TOF analysis of TAXI-I contained in extracellular fluids of *Arabidopsis* transgenic lines; Table S2: list of primers used in this work.
