# Supplementary material for: The Xylanase Inhibitor TAXI-I Increases Plant Resistance to Botrytis cinerea by Inhibiting the BcXyn11a Xylanase Necrotizing Activity
Source: Plants (Basel). 2020 May 8;9(5):601. doi: 10.3390/plants9050601 (PMC7285161; doi:10.3390/plants9050601)
Supplement: Supplementary file 1 [file plants-09-00601-s001.zip › plants-779151-supplementary/Supplementary Table S1.docx]

| Accession | Description | Score | Coverage | # Proteins | # Unique Peptides |
| --- | --- | --- | --- | --- | --- |
| Q8H0K8 | Xylanase inhibitor OS=Triticum aestivum OX=4565 GN=xiI PE=1 SV=1 - [Q8H0K8_WHEAT] | 1225.19 | 38.31 | 1 | 9 |
|  | A2 | Sequence | # PSMs | # Proteins | # Protein Groups |
|  | High | VNVGVLAAcAPSK | 1 | 1 | 1 |
|  | High | AVEAVAPFGVcYDTK | 1 | 1 | 1 |
|  | High | GSTGVAGLANSGLALPAQVASAQK | 5 | 1 | 1 |
|  | High | VPVPEGALATGGVmLSTR | 5 | 1 | 1 |
|  | High | GGSPAHYISAR | 8 | 1 | 1 |
|  | High | QGTAcVAFVEmK | 2 | 1 | 1 |
|  | High | APAVILGGAQmEDFVLDFDmEK | 15 | 1 | 1 |
|  | High | TLGNNLGGYAVPNVQLGLDGGSDWTmTGK | 1 | 1 | 1 |
|  | High | LPHFTGcGGL | 1 | 1 | 1 |

**Supplementary Table S1** MALDI-TOF/TOF analysis of TAXI-I contained in extracellular fluids of *Arabidopsis* transgenic lines
