# Supplementary material for: The Xylanase Inhibitor TAXI-I Increases Plant Resistance to Botrytis cinerea by Inhibiting the BcXyn11a Xylanase Necrotizing Activity
Source: Plants (Basel). 2020 May 8;9(5):601. doi: 10.3390/plants9050601 (PMC7285161; doi:10.3390/plants9050601)
Supplement: Supplementary file 1 [file plants-09-00601-s001.zip › plants-779151-supplementary/Supplementary Table S2 new.docx]

**Supp1ementary Table S2** List of primers used in this work. Restriction enzyme adaptors are indicated in italics.

| **PRIMER NAME** | **PRIMER SEQUENCE (5’-3’)** | **AMPLICON SIZE (bp)** |
| --- | --- | --- |
| **Primers used for heterologous expression of *B. cinerea* Xyn11a** | | |
| BcXyn11F | ATGGTTTCTGCATCTTCCCT | 684 |
| BcXyn11R | TTAAGAAACAGTGATGGAAGCGG |  |
| BcXyn11F+A | *ATGCAGAATTCG*CGCCAGCCGCGGCAC | 627 |
| BcXyn11R+A | *ATGCATCTAGA*TTAAGAAACAGTGATGGAAGCGG |  |
| **Primers for cloning TAXI-I gene** | | |
| TAXI-I_1F_BamHI | *AATAGGATCC*ATGCCACCAGTGCTCCTCCTC | 1208 |
| TAXI-I_1259R SacI | *AATAGAGCTC*TTACAGGCCGCCGCAACC |  |
| **Primers used for PCR screening and gene expression analysis of transgenic plants** | | |
| TAXI-I_774F | CTACCCTACGTCTTGCTCCG | 248 |
| TAXI-I_1002R | TCCACCATCGAGTTCTTCCC |  |
| UBQ5-F | GTGGTGCTAAGAAGAGGAAGA | 251 |
| UBQ5-R | TCAAGCTTCAACTCCTTCTTT |  |
| Actin Tob103-For | TAGGCTGGATTTGCTGGTGA | 196 |
| Actin Tob103-Rev | TCCATGTCATCCCAGTTGCT |  |
