# Supplementary figures and images for: The Xylanase Inhibitor TAXI-I Increases Plant Resistance to Botrytis cinerea by Inhibiting the BcXyn11a Xylanase Necrotizing Activity
Source: Plants (Basel). 2020 May 8;9(5):601. doi: 10.3390/plants9050601 (PMC7285161; doi:10.3390/plants9050601)

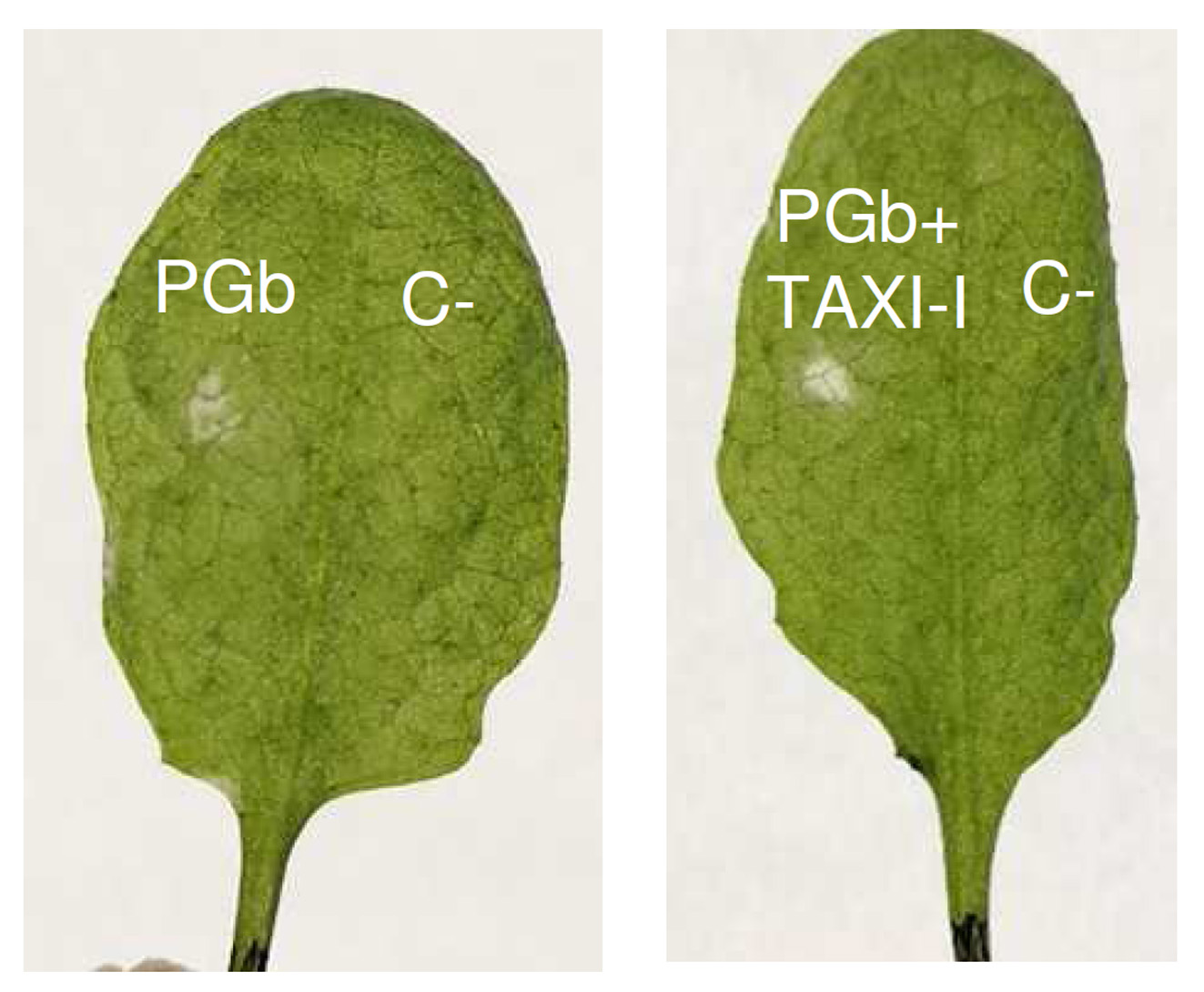

Supplement: Supplementary file 1 [file plants-09-00601-s001.zip › plants-779151-supplementary/Supplementary Figure S1.jpg]

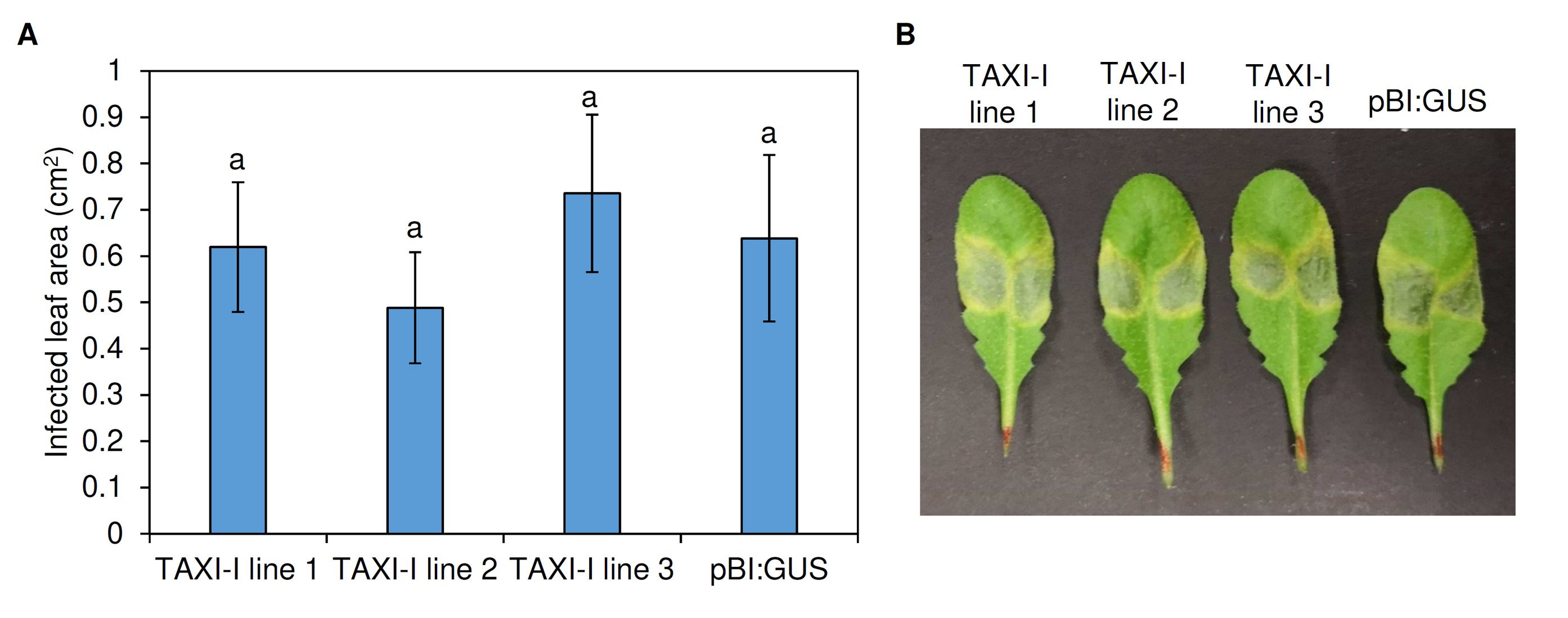

Supplement: Supplementary file 1 [file plants-09-00601-s001.zip › plants-779151-supplementary/Supplementary Figure S2.jpg]

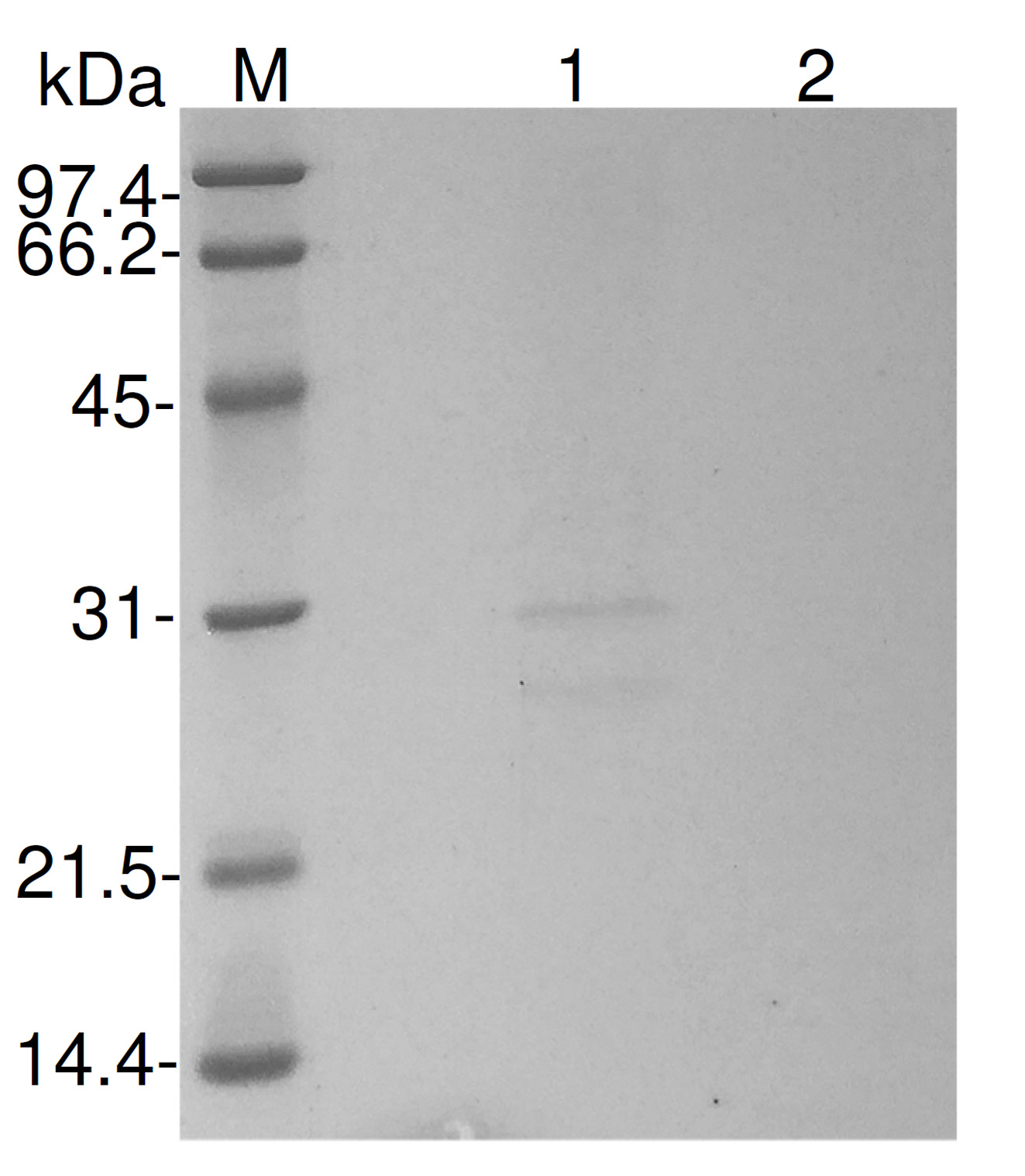

Supplement: Supplementary file 1 [file plants-09-00601-s001.zip › plants-779151-supplementary/Supplementary Figure S3.jpg]

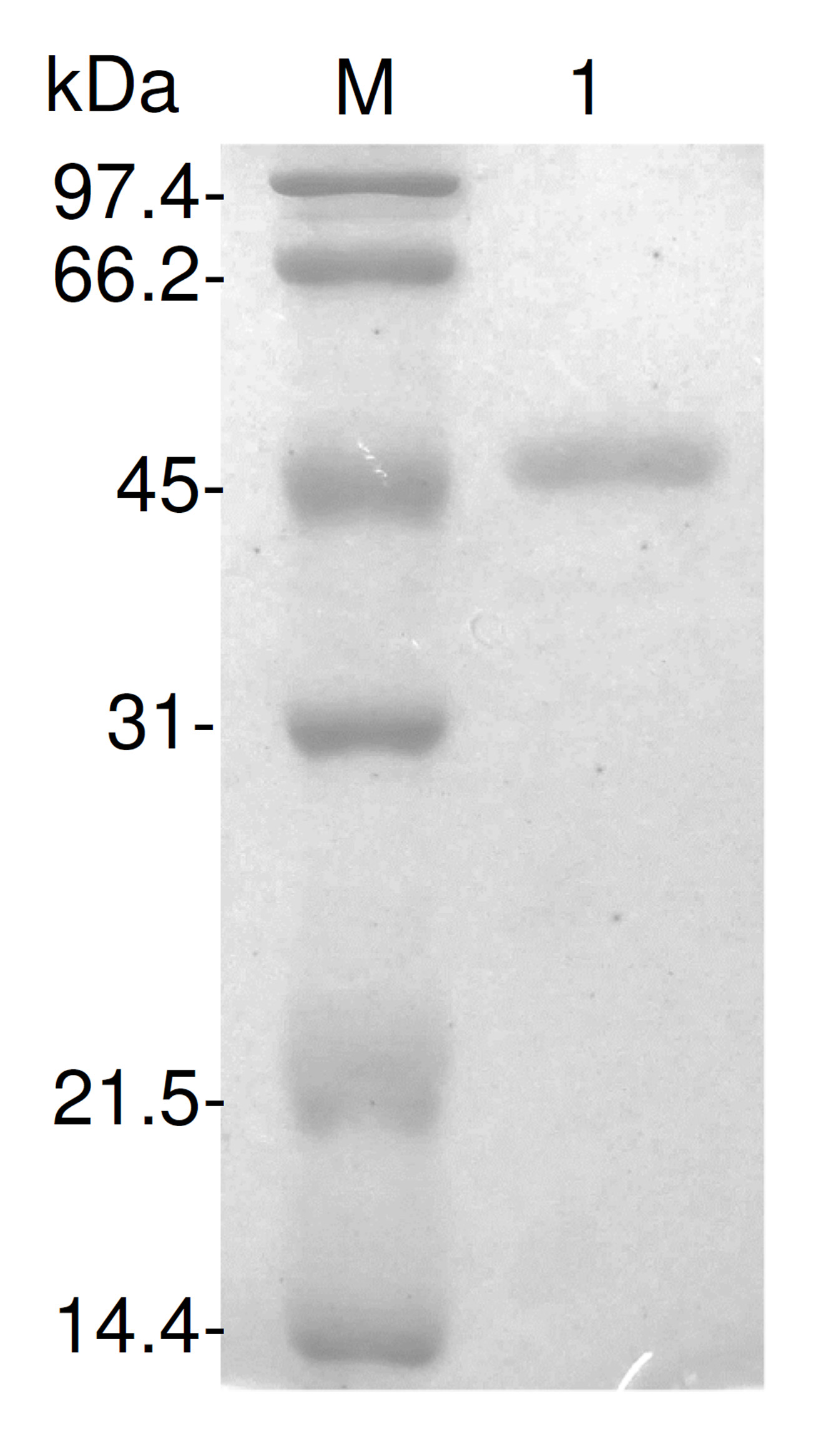

Supplement: Supplementary file 1 [file plants-09-00601-s001.zip › plants-779151-supplementary/Supplementary Figure S4.jpg]
